# Present geothermal field of the Santos Basin, Brazil

**DOI:** 10.1038/s41598-023-39702-5

**Published:** 2023-07-31

**Authors:** Guoping Zuo, Hongping Wang, Lei Lan, Yonggang Zhang, Yinhui Zuo, Liu Yang, Chaofeng Wang, Xu Pang, Xu Song, Meihua Yang

**Affiliations:** 1 PetroChina Hangzhou Research Institute of Geology, Hangzhou, 310023 China; 2 State Key Laboratory of Oil and Gas Reservoir Geology and Exploitation, Chengdu University of Technology, Chengdu, 610059 China

**Keywords:** Geology, Structural geology

## Abstract

The Santos Basin, located in the southeastern waters of Brazil, is a passive continental margin basin with the most abundant deepwater petroleum resources in the world discovered to date. However, few studies have been conducted on the present geothermal fields of the Santos Basin, which severely restricts the oil and gas resource evaluation of the basin. This study first utilizes 35 temperature data from 16 post-salt drilling wells and 370 temperature data from 31 pre-salt drilling wells to calculate the post-salt and pre-salt geothermal gradients and terrestrial heat flows in the Santos Basin. Then, the basin simulation software BasinMod 1D is used to quantitatively evaluate the impacts of salt rock sedimentation on the present geothermal fields and the maturity of pre-salt hydrocarbon source rocks. The results demonstrate that the present post-salt geothermal gradient in the Santos Basin is 2.20–3.97 °C/100 m, with an average value of 2.99 °C /100 m, and the post-salt terrestrial heat flow is 54.00–97.32 mW/m^2^, with an average value of 73.36 mW/m^2^, while the present pre-salt geothermal gradient is 2.21–2.95 °C/100 m, with an average value of 2.53 °C/100 m, and the pre-salt terrestrial heat flow is 61.85–82.59 mW/m^2^, with an average value of 70.69 mW/m^2^. These values are characteristic of a low-temperature geothermal field in a zone with a stable structure. The sedimentation of the salt rock causes a decrease in the temperature of the pre-salt strata, which inhibits pre-salt hydrocarbon source rock maturity, with an inhibition rate of up to 1.32%. The inhibition degree decreases with increasing salt rock thickness. At the same time, the salt rock thickness is positively correlated with the present surface heat flow. The unique distribution of the salt rock and related salt structures lead to present terrestrial heat flow differences among different structural units in the basin. This study is of great significance for evaluating and exploring the pre-salt oil and gas resources in the Santos Basin.

## Introduction

The geothermal fields play an important role in the generation of oil and gas in basin evolutions^1–3^. Studying the geothermal fields in basins has important guiding significance for oil and gas exploration and provides a scientific basis and constraints for exploring basic issues regarding basins, such as genetic dynamics and thermal history construction. The present geothermal fields are the final stage in the evolution of the paleo geothermal fields, which can provide basic geothermal data for the thermal history construction and genetic dynamics of basins, as well as a basis for evaluating oil and gas resources. The present geothermal fields in basins are studied mainly via geothermal methods and techniques. Exploring the present geothermal state of a basin and the lithosphere scale involves studying temperature variations with depth and the spatial distribution characteristics of the geothermal gradient and terrestrial heat flow.

The Santos Basin in Brazil is a passive continental margin basin with the most abundant deepwater petroleum resources in the world discovered to date^4^. Most oil and gas resources have been discovered concentrated beneath thick layers of salt rocks in the Sao Paulo plateau of the deepwater area^5^. Since the discovery of the Lula Oilfield in 2006, Petrobras has increased investment in exploring and developing pre-salt resources in the deepwater area of the Santos Basin. By the end of 2021, a total of 38 oil and gas fields in pre-salt layers had been discovered, including seven giant fields with geological reserves of more than 10 × 10^8^ tons. The cumulative proven oil and gas reserves are approximately 60 × 10^8^ tons of oil equivalent, accounting for 97% of the total proven reserves in the basin, demonstrating the abundant oil and gas resources and immense exploration potential of the pre-salt layers in the basin^6^.

Geothermal measurements have been performed for more than 1360 areas in the southeastern waters of Brazil and the geothermal gradients and terrestrial heat flow values of different sedimentary basins were obtained^7–9^. Hamaza et al. (2017) calculated the geothermal gradients and terrestrial heat flow values of the sedimentary basins along the Brazilian continental margin using corrected bottom hole temperature data and thermophysical parameters of rocks. The average geothermal gradient of the Santos Basin is 2.48 °C/100 m, and the average terrestrial heat flow is 70 mW/m^2^ ^10^. However, previous studies on the present geothermal field in the Santos Basin did not distinguish between the post-salt strata, salt rock layers, and pre-salt strata. Additionally, the thermal effect of salt rocks on the present geothermal fields was not considered resulting in an insufficient understanding of the thermal state of the study area.

Strong tectonic movements, volcanic activity, strata sedimentation, and stratigraphic structures can cause sustained changes in subsurface temperatures, thereby affecting the present geothermal fields. The salt rocks have special thermophysical properties including high thermal conductivity and a low heat production rate. The thermal conductivity of salt rocks can reach 6 W/(m·K), which is 2–3 times that of ordinary sedimentary rocks, and the heat production rate can be as low as 0.01–0.23 μW/m^3^, only 1/20 to 1/30 of that of ordinary sedimentary rocks^11^. The heat from the pre-salt strata can be conducted through the salt rock to the post-salt strata, resulting in a hot blanket effect and causing higher temperature on the post-salt strata or the flanks of salt domes. The Santos Basin is characterized by a thick layer of salt rocks, with the maximum thickness exceeding 2,000 m in the eastern uplift areas and reaching up to 2,500 m in some local areas^12^. This study is based on temperature data from 16 post-salt drilling wells and 31 pre-salt drilling wells in the Santos Basin. Combined with logging data, the post-salt and pre-salt geothermal gradients and terrestrial heat flow in the Santos Basin are calculated separately to identify the characteristics of the present geothermal field in the Santos Basin, which can provide important parameters for evaluating pre-salt oil and gas resources in the Santos Basin, and provide basic geothermal parameters for the evaluation of post-salt and pre-salt geothermal resources.

## Geological settings

**Figure 1:**
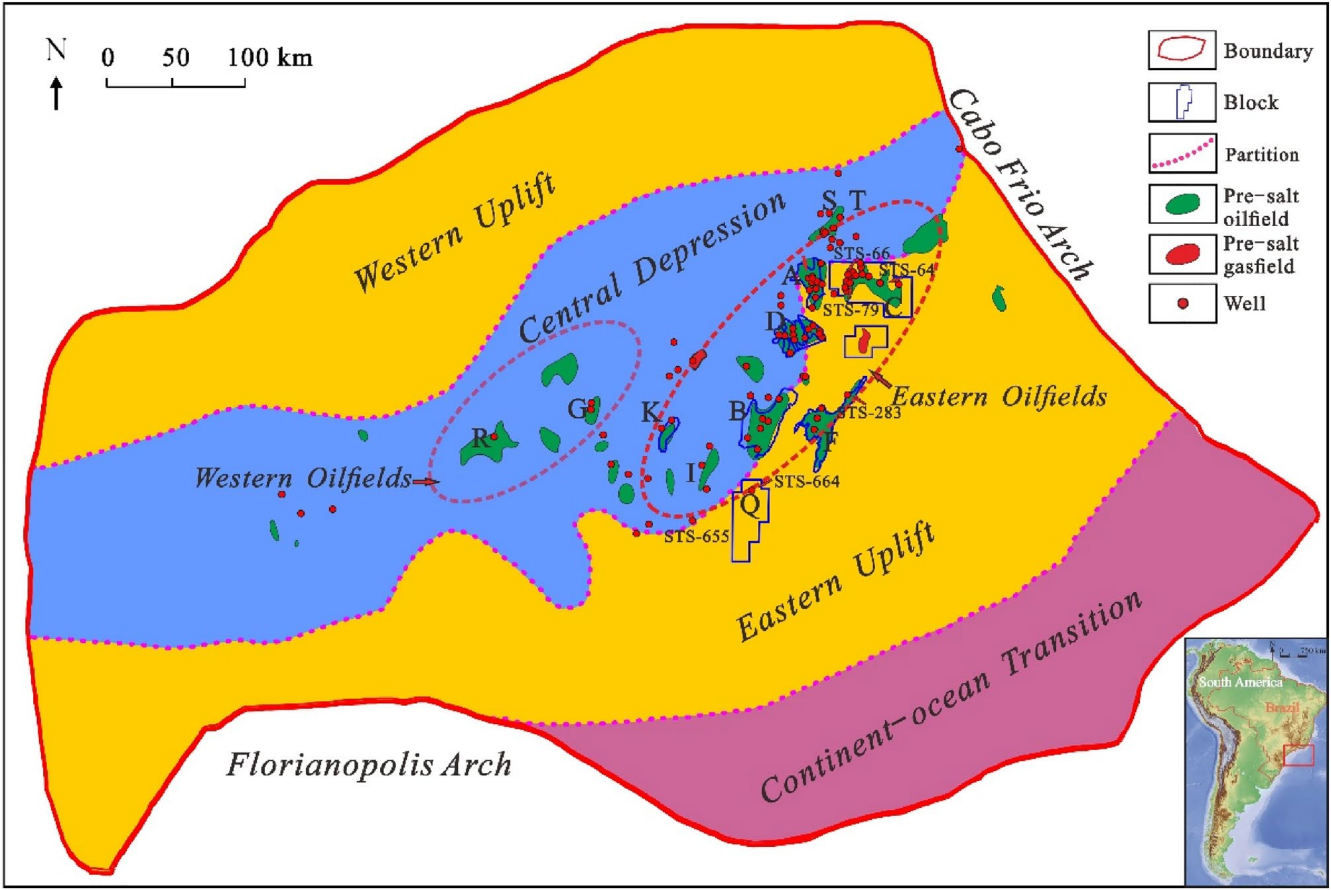
Tectonic divisions and distribution of oil and gas fields in the Santos Basin. Figures were produced by CorelDRAW Graphics Suite X8 (https://www.corel.com/en/) and 91weitu v19.3.4 (https://www.91weitu.com).

The Santos Basin is located in the southeastern waters of Brazil (23°00'–28° 30' S, 39° 30'–48° 30' W), bordered in the northeast by the Campos Basin at the Cabo Frio Arch and in the southwest by the Pelotas Basin at the Florianopolis Arch. It has an area of approximately 327,000 km^2^ and a water depth of 0 to 3,200 m^13–18^. The pre-salt oil and gas are distributed mainly in the Central Depression Zone and the Eastern Uplift Zone (Fig. 1). The Santos Basin, together with the Campos Basin and the Espirito Santo Campos Basin in the north forms the Great Campos Basin. These three basins are typical passive continental margin basins with similar structural evolution and sedimentation filling histories and have excellent petroleum geological conditions and many discovered oil and gas reserves^19^.

**Figure 2:**
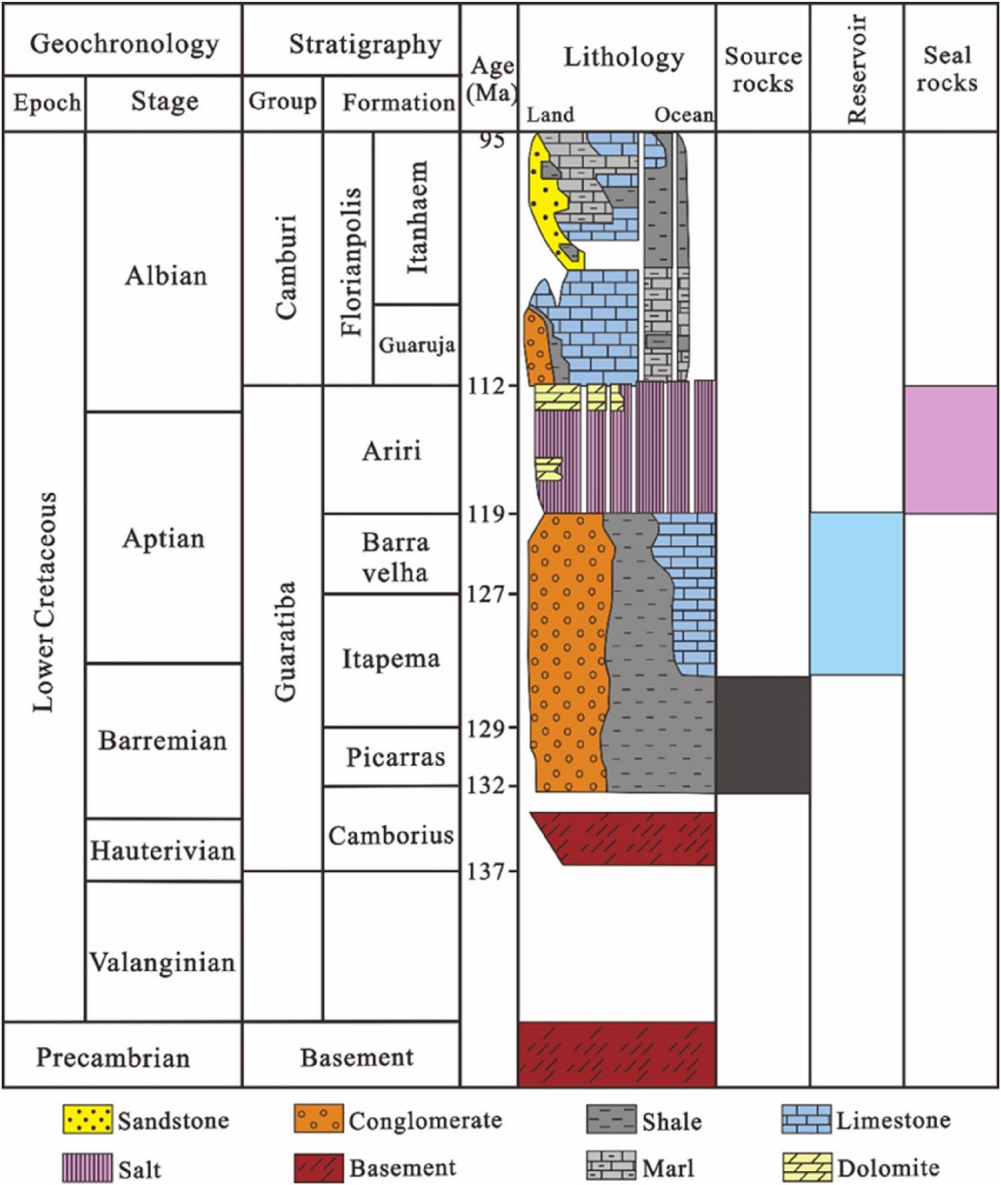
Stratigraphic column of the Santos Basin.

The Santos Basin formed during the breakup of the Gondwana continent and the opening of the South Atlantic^20^. As the South Atlantic rifted from north to south, the Santos basin experienced four stages of tectonic evolution including a pre-rift stage, a rift stage, a transitional stage, and a passive continental margin stage. Before the Late Jurassic, at the pre-rift intracratonic stage, there was intense volcanic activity, mainly consisting of alluvial fan systems in a dry climate. During the Early Cretaceous, in the Berriasian-early Aptian rift stage, the ancient continent split from south to north, forming a series of grabens and horsts in the basin under extensional forces. The late Aptian in the Early Cretaceous was a sagging stage in which the basin entered a thermal subsidence period characterized by a dry and limited sedimentary environment. During the Albian period of the Cenozoic, as the plates continued to split, seawater flooded in, and the Santos basin entered a drifting stage, gradually evolving from a shallow-water carbonate rock platform sedimentary environment to an ocean environment with an open passive continental margin^21,22^. The Santos Basin was formed on top of a pre-Cambrian crystalline basement and developed from bottom to top with the following components: volcanic rocks of the Early Cretaceous Camboriu Formation, lacustrine sandstones and shales of the Early Cretaceous Guaratiba Formation, salt rocks of the Ariri Formation, marine sandstones of the Florianopolis Formation, carbonate rocks of the Guaruja Formation, calcilutite of the Itanhaem Formation, mudstones and turbidite sandstones of the Itajai-Acu, Santos, and Jureia Formations, as well as turbidite sandstones, mudstones, and carbonate rocks of the Marambaia, Iguape, and Sepetiba Formations (Fig. 2)^23^.

## Methods and parameters

### Geothermal gradient calculation method

The thermal conductivity of salt rock is considerably greater than that of ordinary sedimentary rock (approximately 2–3 times), and salt rock sedimentation can cause changes in the post-salt and pre-salt geothermal gradients^24^. Therefore, Eqs. 1 and 2 are used to calculate the geothermal gradients of the post-salt and pre-salt strata, respectively.

1 $$G_{post} = \left( {T_{1} {-\!\!-}T_{0} } \right)/Z_{1}$$

where *G*_*post*_ is the geothermal gradient of the post-salt strata, °C/100 m; *T*_*1*_ is the temperature of the post-salt strata, °C; *T*_*0*_ is the temperature of the bottom of the sea, 2 °C; and *Z*_*1*_ is the depth of the strata, m.

2 $$G_{pre} = \left( {T_{3} {-\!\!-}T_{2} } \right)/\left( {Z_{3} {-\!\!-}Z_{2} } \right)$$

where *G*_*pre*_ is the geothermal gradient of the pre-salt strata, °C/100 m; *T*_*3*_ is the temperature of the pre-salt strata, °C; *T*_*2*_ is the temperature on top of the pre-salt strata, °C; *Z*_*3*_ is the depth of the pre-salt strata, m; and *Z*_*2*_ is the top depth of the pre-salt strata, m.

### Terrestrial heat flow calculation method

Terrestrial heat flow is a comprehensive parameter that can reflect the characteristics of a geothermal field in a region more accurately than other geothermal parameters, such as temperature and geothermal gradient. It can be calculated using the following equation:

3 $$q = - K \times G$$

where *q* represents the terrestrial heat flow, mW/m^2^; K represents the rock thermal conductivity, W/(m·K); G represents the geothermal gradient, °C/km; and the negative sign indicates that the direction of terrestrial heat flow is opposite to that of the geothermal gradient.

### Basic parameters

**Figure 3:**
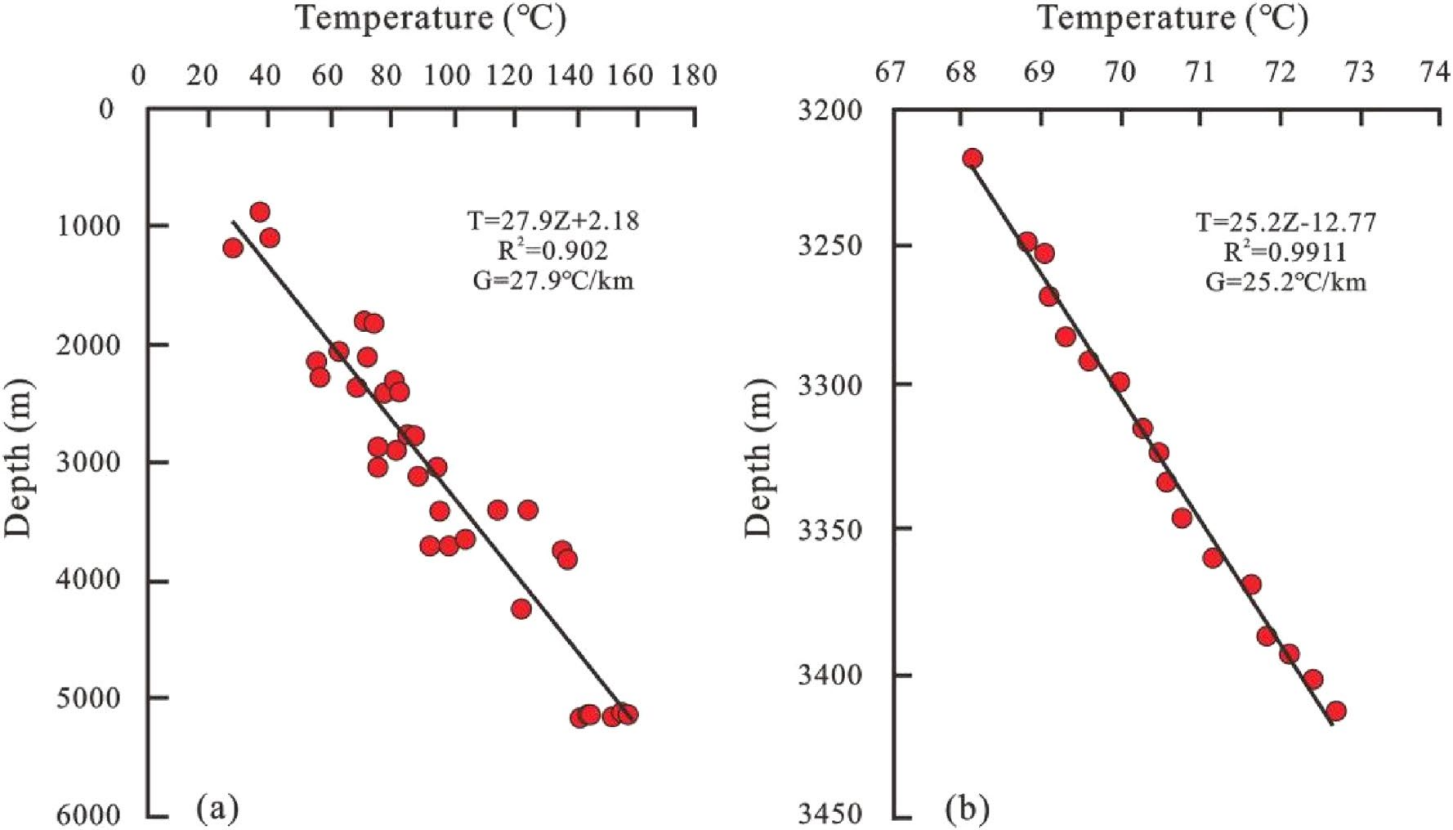
(**a**) Linear relationship between pre-salt temperature and depth; (**b**) Linear relationship between post-salt temperature data and depth.

The temperature data includes 35 bottom hole temperature data from 16 post-salt wells and 370 bottom hole temperature data from 31 pre-salt wells. The post-salt temperature data are concentrated in the STS-S and STS-T blocks, whereas the pre-salt temperature data are distributed mainly in the STS-C, STS-D, and STS-A blocks, with a small amount of data in the STS-F and STS-B blocks. Taking the STS-374 well in the STS-A block as an example, the pre-salt and post-salt temperature data exhibit a good linear relationship with the depth, which suggests that the geothermal field of the Santos Basin has the characteristic of thermal conduction (Fig. 3). Due to the lack of rock thermal conductivity data for the Santos Basin, thermal conductivity data from the nearby Campos Basin are used^9^.

## Results

The present geothermal gradient of the post-salt in the Santos Basin is 2.20–3.97 °C/100 m, with an average value of 2.99 °C/100 m, and the terrestrial heat flow is 54.00–97.32 mW/m^2^, with an average value of 73.36 mW/m^2^. The present geothermal gradient of the pre-salt ranges from 2.21 to 2.95 °C/100 m, with an average value of 2.53 °C/100 m. The terrestrial heat flow ranges from 61.85 to 82.59 mW/m^2^, with an average value of 70.69 mW/m^2^. In the STS-D block, the present geothermal gradient ranges from 2.44 to 2.64 °C/100 m, with an average value of 2.55 °C/100 m, and the terrestrial heat flow ranges from 68.42 to 73.86 mW/m^2^, with an average value of 71.23 mW/m^2^. In the STS-A block, the present geothermal gradient ranges from 2.32 to 2.67 °C/100 m, with an average value of 2.48 °C/100 m, and the terrestrial heat flow ranges from 64.93 to 74.81 mW/m^2^, with an average value of 69.53 mW/m^2^. In the STS-F block, the present geothermal gradient has an average value of 2.38 °C/100 m, and the terrestrial heat flow has an average value of 66.6 mW/m^2^. In the STS-C block, the present geothermal gradient ranges from 2.25 to 2.95 °C/100 m, with an average value of 2.55 °C/100 m, and the terrestrial heat flow ranges from 62.97 to 82.59 mW/m^2^, with an average value of 71.25 mW/m^2^. In the northern area of the STS-C block, the present geothermal gradient ranges from 2.43 to 2.65 °C/100 m, with an average value of 2.54 °C/100 m, and the terrestrial heat flow ranges from 68.05 to 74.25 mW/m^2^, with an average value of 71.15 mW/m^2^. In the STS-B block, the present geothermal gradient has an average value of 2.75 °C/100 m, and the terrestrial heat flow has an average value of 76.94 mW/m^2^. In the STS-R block, the present geothermal gradient has an average value of 2.43 °C/100 m, and the terrestrial heat flow has an average value of 68.00 mW/m^2^ (Table 1). Overall, the Santos Basin has a low-temperature geothermal field, as in a tectonically stable area, and the geothermal gradient of the post-salt strata is slightly higher than that of the pre-salt strata. The thermal conductivity of the rock above the salt layer is lower than that of the rock below the salt layer, and the thermal conductivity of the salt rock is higher, the heat inside the earth is rapidly transmitted to the rock above the salt layer through the salt layer, resulting in the geothermal gradient above the salt layer higher than the geothermal gradient below the salt layer.

**Table 1:** Computation of geothermal gradient and terrestrial heat flow in the Santos Basin.

Block	Well	G ( °C/100 m)	q (mW/m^2^)	Horizon	Block	Well	G ( °C/100 m)	q (mW/m^2^)	Horizon
STS-S	STS-131	2.92	71.61	post-salt	STS-A	STS-56	2.32	64.93	Pre-salt
STS-S	STS-155	3.97	97.32	post-salt	STS-Q	STS-664	2.21	61.85	Pre-salt
STS-T	STS-98	2.99	73.33	post-salt	STS-B	STS-543	2.75	76.94	Pre-salt
STS-T	STS-99	2.90	71.07	post-salt	STS-W	STS-406	2.65	74.25	Pre-salt
STS-T	STS-101	2.67	65.47	post-salt	STS-X	STS-403	2.43	68.05	Pre-salt
STS-T	STS-158	3.76	92.21	post-salt	STS-V	STS-405	2.54	71.17	Pre-salt
STS-T	STS-102	2.40	58.86	post-salt	STS-C	STS-428	2.34	65.48	Pre-salt
STS-T	STS-104	2.84	69.60	post-salt	STS-C	STS-440	2.42	67.72	Pre-salt
STS-T	STS-139	2.95	72.35	post-salt	STS-C	STS-442	2.36	66.06	Pre-salt
STS-T	STS-140	2.59	63.54	post-salt	STS-C	STS-79	2.25	62.97	Pre-salt
STS-T	STS-141	2.49	61.05	post-salt	STS-C	STS-92	2.67	74.72	Pre-salt
STS-U	STS-109	2.90	70.94	post-salt	STS-C	STS-88	2.90	81.04	Pre-salt
STS-V	STS-110	3.52	86.16	post-salt	STS-C	STS-67	2.63	73.72	pre-salt
/	STS-143	3.33	81.51	post-salt	STS-C	STS-89	2.75	76.86	Pre-salt
/	STS-160	3.45	84.63	post-salt	STS-C	STS-81	2.95	82.59	Pre-salt
/	STS-181	2.20	54.00	post-salt	STS-C	STS-666	2.77	77.52	Pre-salt
STS-D	STS-227	2.64	73.86	pre-salt	STS-C	STS-68	2.62	73.32	Pre-salt
STS-D	STS-271	2.53	70.94	pre-salt	STS-C	STS-94	2.32	64.94	Pre-salt
STS-D	STS-286	2.57	71.84	pre-salt	STS-C	STS-667	2.31	64.65	Pre-salt
STS-D	STS-307	2.56	71.54	pre-salt	STS-C	STS-93	2.58	72.20	Pre-salt
STS-D	STS-220	2.44	68.42	pre-salt	STS-C	STS-80	2.32	64.90	Pre-salt
STS-D	STS-277	2.53	70.81	pre-salt	STS-F	STS-283	2.38	66.60	Pre-salt
STS-A	STS-374	2.67	74.81	pre-salt	STS-R	STS-665	2.43	68.00	Pre-salt
STS-A	STS-136	2.46	68.84	pre-salt					

## Discussion

### Impact of salt rocks on the present geothermal fields

By calculating the post-salt and pre-salt geothermal gradients and terrestrial heat flows in the Santos Basin, this study reveals that the Santos Basin has the characteristic of a low-temperature geothermal field, as in a tectonically stable zone. The post-salt geothermal gradient (2.99 °C/100 m) is higher than the pre-salt geothermal gradient (2.53 °C/100 m), while the post-salt terrestrial heat flow value (73.4 mW/m^2^) is equivalent to the pre-salt value (70.7 mW/m^2^). The Santos Basin generally contains thick salt rocks, and the formation of oil and gas reservoirs is closely related to these salt layers. The strong contrast in thermophysical properties between the salt rocks and surrounding strata will inevitably cause changes in the thermal regime of the basin. Under the condition of steady-state heat conduction, the influence of salt rock sedimentation on the post-salt and pre-salt formation temperatures and geothermal gradients is simulated. The results demonstrate that the temperature of the overlying layer of the salt body in the salt rock sedimentation area increases by 17–25 °C and that a strong local geothermal gradient forms^25^. At the same time, the sedimentation of salt rocks can also suppress the thermal evolution of the pre-salt hydrocarbon source rocks^11,26–32^.

**Figure 4:**
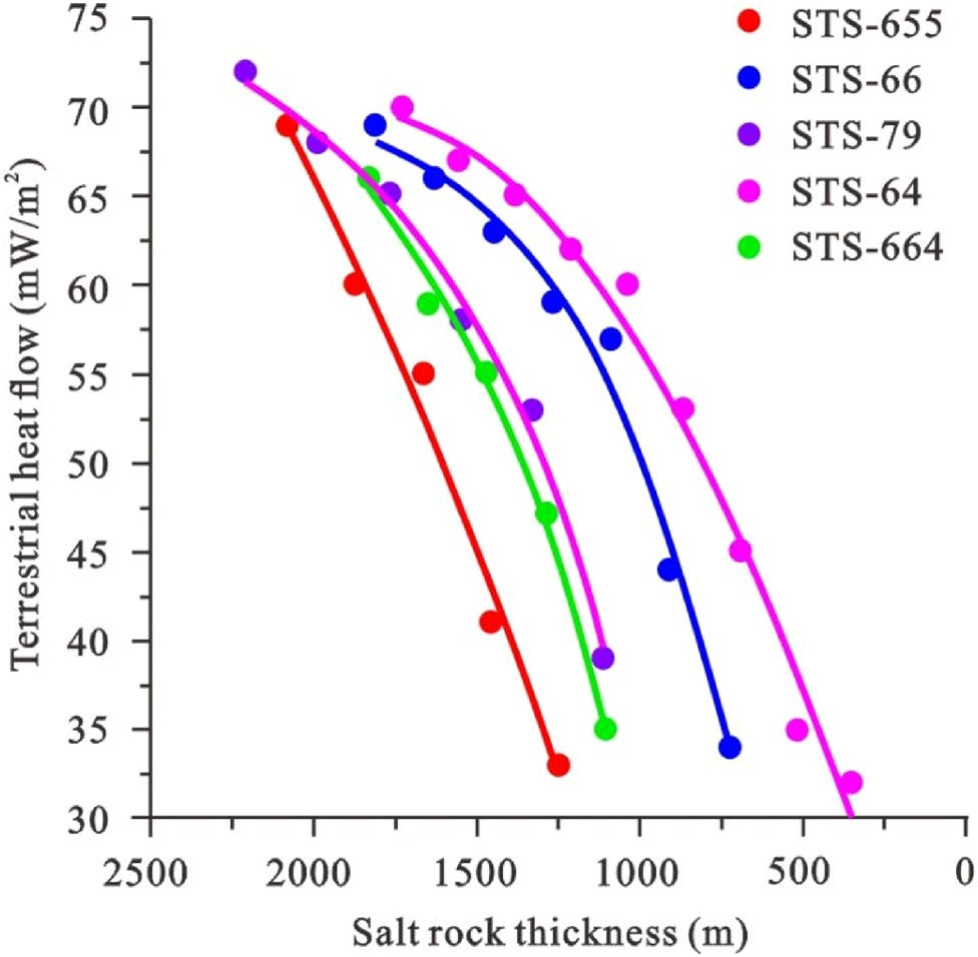
Influence of salt rock thickness on surface heat flow.

This study considers the five individual wells in the Santos Basin as examples. While ensuring that the basic geological parameters (including the stratified data, rock thermal conductivity, and heat production rate) remain unchanged, the impact of salt rock sedimentation on the present geothermal field is analyzed by reducing the salt thickness by 10%. In addition, the measured vitrinite reflectance is used to correct the thermal history (Table 2). The results demonstrate that as the thickness of the salt rock gradually decreases, the surface heat flow dwindles (Fig. 4).

**Table 2:** Influence of salt rock thickness on surface heat flow.

STS-655		STS-66		STS-79		STS-664		STS-283		STS-64	
Thickness(m)	*q* (mW/m^2^)	Thickness(m)	*q* (mW/m^2^)	Thickness(m)	*q* (mW/m^2^)	Thickness (m)	*q* (mW/m^2^)	Thickness (m)	*q* (mW/m^2^)	Thickness (m)	*q* (mW/m^2^)
2080	69	1813	69	2211	72	1832	66	316	49	1730	70
1872	60	1631	66	1990	68	1649	59	284	47	1557	67
1664	55	1450	65	1769	65	1466	55	253	45	1384	65
1456	41	1269	59	1548	58	1282	47	221	44	1211	62
1248	33	1088	57	1327	53	1099	35	190	43	1038	60
1040	–	906	45	1106	39	916	20	158	42	865	53
832	–	725	34	884	24	733	–	126	41	692	45

### Tectonic settings of the terrestrial heat flow

The Santos Basin is currently in a passive continental margin stage, with low tectonic activity^10^. It is undergoing a relatively stable thermal subsidence^33,34^. Since the pre-rift stage, the basin has undergone multiple instances of volcanic activity. The most recent volcanic activity occurred approximately 70–90 million years ago. However, the thermal disturbance and relaxation time on the scale of the lithosphere is approximately 62 million years^35^. Since the passive continental margin stage began, the Santos Basin has not been affected by large-scale magmatic activity. Therefore, the thermal anomaly caused by magmatic activity in the Santos Basin has no influence on the present geothermal field. Due to the strong fluidity and easy deformability, the salt rocks of the Ariri Formation form various salt structures during the basin sedimentation process under the influence of sediment progradation, overlying strata stretching, gravity sliding, gravitational expansion, and possible underlying rift valley structures^23^. The unique distribution of salt rocks and the related salt structures lead to the terrestrial heat flow at the present day are different among different structural units within the basin.

### Influence of salt rocks on the maturity of pre-salt source rocks

**Figure 5:**
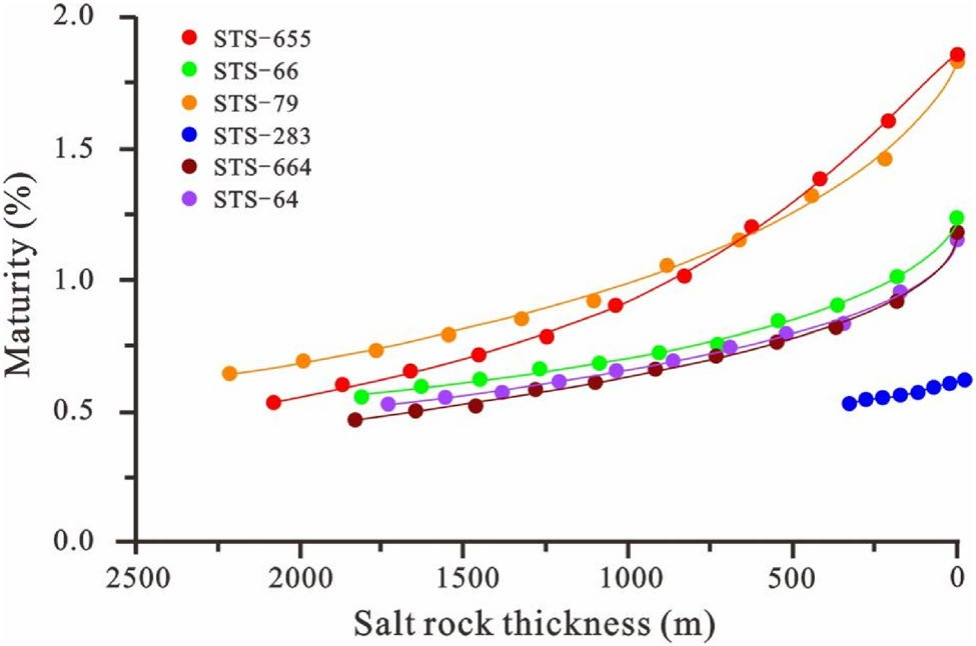
Influence of salt rock thickness on top maturity of the Itapema Formation.

To characterize the influence of the salt rock with high thermal conductivity on the maturity of the pre-salt strata, this study reduces the salt rock thickness by 10% while setting the basic geological parameters of six individual wells (such as the stratified data, rock thermal conductivity, and rock heat production rate) and the thermal history as a constant (Table 3). Then the relationship between salt rock thickness and maturity is studied using the basin simulation software BasinMod 1D. The results demonstrate that after salt rock sedimentation, the temperature of the pre-salt strata is reduced due to the influence of salt rock sedimentation, thereby inhibiting its maturity by up to 1.32%. However, as the salt rock thickness increases, the inhibitory effects of the salt rock on the temperature and maturity of the pre-salt strata gradually weaken (Fig. 5).

**Table 3:** Influence of salt rock thickness on top maturity of the Itapema Formation.

Proportion of salt rock (%)	STS-664		STS-283		STS-655		STS-66		STS-64		STS-79	
	Thickness (m)	R_o_ (%)	Thickness (m)	R_o_ (%)	Thickness (m)	R_o_ (%)	Thickness (m)	R_o_ (%)	Thickness (m)	R_o_ (%)	Thickness (m)	R_o_ (%)
100	1832	0.46	316	0.53	2080	0.53	1813	0.55	1730	0.52	2211	0.64
90	1649	0.50	284	0.54	1872	0.60	1631	0.59	1557	0.55	1990	0.69
80	1466	0.52	253	0.55	1664	0.65	1450	0.62	1384	0.57	1769	0.73
70	1282	0.58	221	0.55	1456	0.71	1269	0.66	1211	0.61	1548	0.79
60	1099	0.61	190	0.56	1248	0.78	1088	0.68	1038	0.65	1327	0.85
50	916	0.66	158	0.56	1040	0.90	906	0.72	865	0.69	1106	0.92
40	733	0.71	126	0.57	832	1.01	725	0.75	692	0.74	884	1.05
30	549	0.76	95	0.58	624	1.20	544	0.84	519	0.79	663	1.15
20	366	0.82	63	0.59	416	1.38	363	0.90	346	0.83	442	1.32
10	183	0.92	32	0.60	208	1.60	181	1.01	173	0.95	221	1.46
0	0	1.18	0	0.61	0	1.85	0	1.23	0	1.15	0	1.83

## Conclusions

(1) The post-salt geothermal gradient in the Santos Basin is 2.20 ~ 3.97 °C/100 m, with an average value of 2.99 °C/100 m, and the terrestrial heat flow is 54.0 ~ 97.3 mW/m^2^, with an average value of 73.4 mW/m^2^. The pre-salt geothermal gradient ranges from 2.21 to 2.95 °C/100 m, with an average value of 2.53 °C/100 m, and the terrestrial heat flow ranges from 61.9 to 82.6 mW/m^2^, with an average value of 70.7 mW/m^2^. Overall, the Santos Basin has a low-temperature geothermal field, as in a tectonically stable zone.

(2) The sedimentation of the salt rock in the Santos Basin has caused a decrease in the temperature of the pre-salt strata, which inhibits the maturity of the pre-salt hydrocarbon source rocks by up to 1.32%. This inhibitory effect decreases with increasing salt rock thickness. In addition, the salt rock thickness is positively correlated with the present surface heat flow. The unique distribution of salt rocks and related salt structures result in present terrestrial heat flow differences among different structural units in the Santos Basin.

## Data Availability

Data are contained in the tables of the article.
